# Neutrophils recruited to immunization sites initiating vaccine-induced antibody responses by locally expressing BAFF

**DOI:** 10.1016/j.isci.2022.104453

**Published:** 2022-05-23

**Authors:** Yangyang Wang, Kuo Qu, Wenting Lu, Peiyan Zhao, Zhe Wang, Cuiyun Cui, Ye Liu, Ming Yang, Yongli Yu, Liying Wang

**Affiliations:** 1 Department of Molecular Biology, College of Basic Medical Sciences and Institute of Pediatrics, First Hospital of Jilin University, Norman Bethune Health Science Center, Jilin University, Changchun, Jilin 130021, China; 2 Department of Immunology, College of Basic Medical Sciences, Norman Bethune Health Science Center, Jilin University, Changchun, Jilin 130021, China

**Keywords:** Components of the immune system, Immune response, Immunology

## Abstract

Neutrophils played a key role in the innate immune responses. Less is known about whether and how the neutrophils recruited in the immunization sites affecting the vaccine-induced antibody responses. In the process of evaluating the efficacy of an oil-in-water emulsion-formulated vaccine in mice, we found that neutrophils were rapidly and massively recruited to immunization sites but were barely detected in the draining lymph nodes. Interestingly, B cell-activating factor (BAFF) was abundantly expressed in the recruiting neutrophils at a very early stage. The initial neutrophil-derived BAFF firstly brought about the B cell responses in the local part, then subsequently in lymphoid organs. Activated B cells produced more BAFF through TLR9-IRF5 signaling pathway, thereby amplifying the vaccine-induced antibody responses. Suppressing BAFF in the neutrophils could weaken the B cell activation and reduce the antibody production. The data indicate that vaccines endow neutrophils with the potential to orchestrate antibody responses at immunization sites.

## Highlights

• Neutrophils at immunization sites influencing subsequent immune responses
• Neutrophil-driven BAFF at immunization sites assisting B cell responses to vaccines
• Activated B cells produce more BAFF through TLR9-IRF5 signaling pathway
• BAFF-producing neutrophils orchestrate antibody responses at immunization sites

## Graphical abstract

**Figure:**
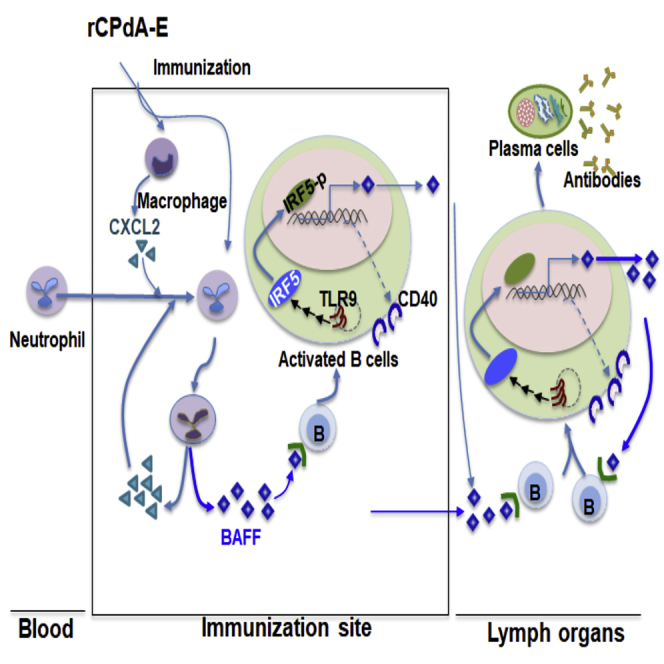

## Introduction

Neutrophils are the first-line effector cells of the innate immune system. When an individual is infected, they are rapidly recruited to the infection site to launch the cascades of innate immune responses, taking up a variety of microorganisms by phagocytosis and releasing the antimicrobial compounds/cytokines (Kolaczkowska and Kubes, 2013). It is worth noting that the rapidly swarmed neutrophils could also be detected around the vaccination sites. Further research is needed to find out whether the neutrophils join and contribute to the complicated adaptive immune responses because of the vaccines (Chorny et al., 2016; Musich et al., 2018; Tchalla et al., 2020; Trentini et al., 2016). Typical vaccines are concoctions of antigens and adjuvants. Aluminum (Alu), oil-in-water emulsions (MF59, AS03 and AF03), and Toll-like receptor 9 (TLR9) agonist CpG oligodeoxynucleotide (CpG ODN) (Givord et al., 2018; Pellegrino et al., 2015) are among the approved vaccine adjuvants of human use. Adjuvants are essential vaccine components that recruit neutrophils in immunization sites. Adjuvant AS03 was reported to recruit neutrophils to the immunization site, where the neutrophils take up antigens and then migrate into the lymph nodes to promote the production of antigen-specific IgG by B cells (Calabro et al., 2011, De Veer et al., 2013, Liang et al., 2017, Puga et al., 2011, Ohtsuka et al., 2001). MF59 formulated vaccines induced massive accumulation of neutrophils in immunization sites and then an efficient transportation of antigens from the immunization site to the DLNs (Calabro et al., 2011). However, few reports are on whether and how the recruited neutrophils in immunization sites participate in activating B cells (Grund et al.,2013, 2017) and therefore enhance the responses of the antigen specific antibody to vaccines.

Presumably, the B cells could be activated by the neutrophil-derived cytokines, because neutrophils produce several B-cell-activating cytokines, including B cell-activating factor (BAFF) (Grund et al., 2013; Scapini et al., 2008), interleukin (IL)-21 (Puga et al., 2011), and IL17A (Grund et al.,2013, 2017). BAFF is synthesized by membrane-bound as well as secreted forms. Besides, it is required for the homeostasis and the activation of the antibody producing B cells (Schneider et al., 1999). B cells constitutively express TLR9 (Cunningham-Rundles et al., 2006). The TLR9 activation makes the B cells to be more sensitive to the effects of BAFF (Abu-Rish et al., 2013). Once being activated, the TLR9 undergoes dimerization, followed by the recruitment and activation of the adaptor protein MyD88 (Suthers and Sarantopoulos, 2017). Consequently, the E3 ubiquitin ligase TNF receptor-associated factor 6 (TRAF6) is recruited, leading to the transcription of the downstream interferon regulatory factor 5 (IRF5) (Suthers and Sarantopoulos, 2017). The IRF5 is required for the activation and proliferation of the B cells at an early stage and for the differentiation of TLR9-induced antibody secreting cells (ASC) (Saurav De et al., 2018). IL-21 activates B cells to proliferate and differentiate into memory B cells and antibody-producing plasma cells (Ettinger et al., 2008, Parrish-Novak et al., 2000). IL-21 gene deletion can markedly reduce the level of serum IgG in mice (Ettinger et al., 2008, Parrish-Novak et al., 2000). Neutrophils were reported to be able to move from the immunization site into marginal zones of spleens and DLNs, where they produce BAFF and IL-21 to activate B cells (Puga et al., 2011; Scapini et al., 2008). Consequently, the paracrine and autocrine BAFF promotes the vigorous production of antigen-specific IgG from B cells (Cerutti et al., 2013; Chorny et al., 2016; Deniset et al., 2017; Puga et al., 2011). Recent studies showed that autoinflammatory neutrophil extracellular traps (NETs) which is composed of self-DNA, neutrophil elastase autoantigen and BAFF could activate B cell response, implying that there is a link between neutrophils and the B cell activation (Jeremic et al., 2019, Lood and Hughes, 2017). Up to now, there are few studies based on whether and how the BAFF-producing neutrophils that are recruited in the immunization site help in activating the B cells in the DLN and the spleen, thereby promoting vaccine-induced antibody responses.

In this study, we found that massively recruited neutrophils in the immunization site could produce BAFF after the i.p. immunization with rCPdA-E, a vaccine formulated with an oil-in-water emulsion. The neutrophil-derived BAFF activated the B cells in the local part and subsequently activated the B cells in the lymphoid organs. The activated B cells amplified the reaction by generating more BAFF through TLR9-IRF5 signaling pathway and therefore led to an enhanced response of the antigen specific antibody to the vaccine. The data reveal an alternative way of understanding how the recruited neutrophils in the immunization site prompt antigen specific antibody responses to vaccines.

## Results

### The neutrophil recruitment in immunization sites positively correlated with vaccine-induced antibody production in mice

**Figure 1:**
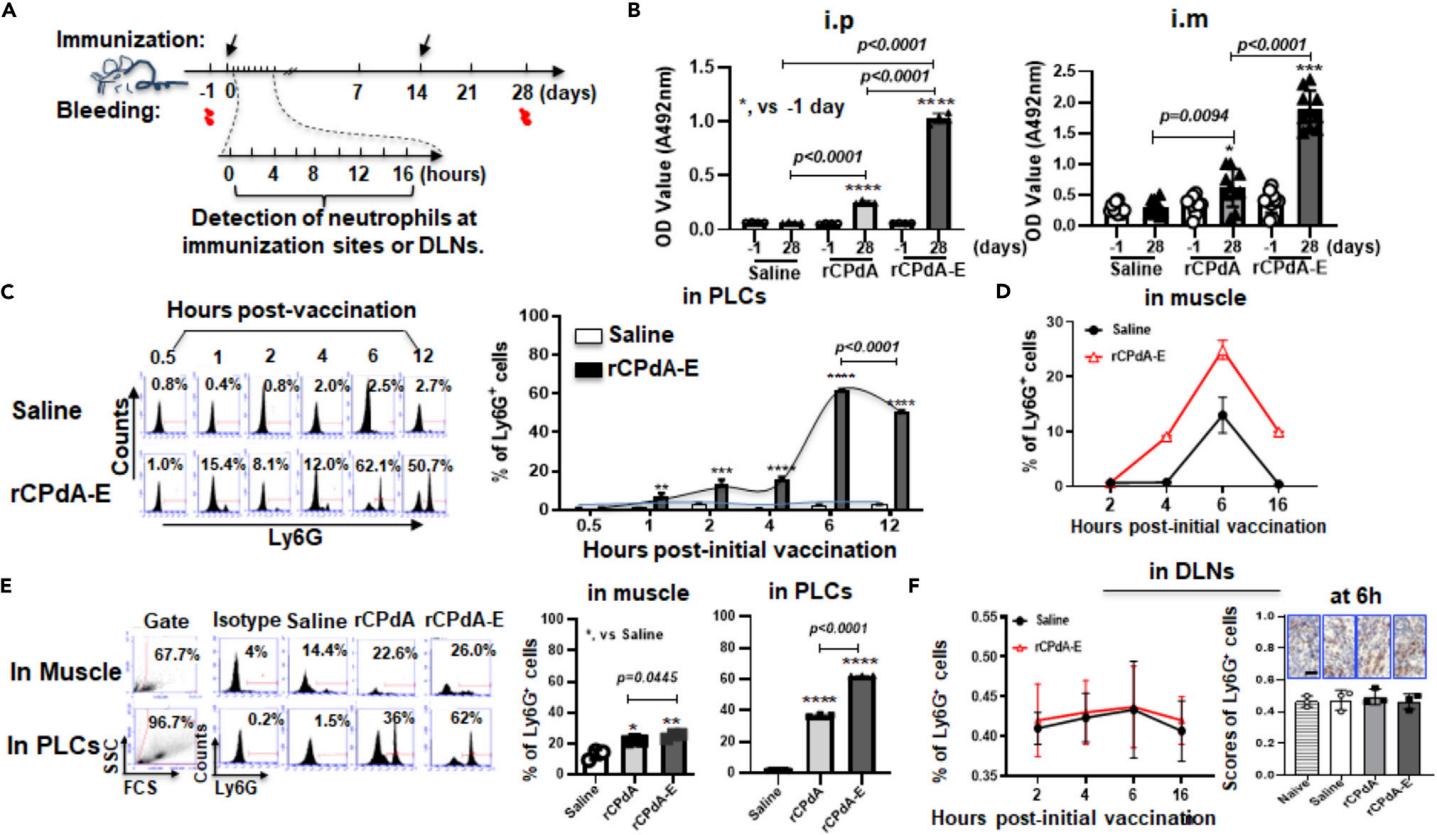
Antibody levels and neutrophil recruitment of immunized mice Mice were immunized with rCPdA or rCPdA-E by intraperitoneal injection (i.p.) or intramuscular injection (i.m.) twice in a 14-day interval for detecting serum antibody by ELISA on day 28 and for detecting the recruitment of neutrophils in immunization sites or DLNs of inguinal lymph nodes at different times post-initial immunization. Saline injected mice were used as negative control. (A) The experiment procedure. (B) The levels of anti-rCPdA antibodies in sera of the immunized mice [n = 3 (i.p.) or n = 10 (i.m.) in each group]. (C) Dynamic percentages of neutrophils in PLCs at the immunization site of rCPdA-E immunized mice (n = 3 in each group) by flow cytometry. (D) Dynamic percentages of neutrophils in muscle tissues at the immunization site of the rCPdA-E immunized mice (n = 3 in each group) by flow cytometry. (E) Percentages of recruited neutrophils in muscle tissues or PLCs of the immunized mice (n = 3 in each group) on 6 h post-initial immunization. (F) Percentages of recruited neutrophils in inguinal lymph nodes of the i.m. immunized mice (n = 3 in each group) at 6 h post-initial immunization. Scale bars represent 20 μm in length. The data were the representative mean values of at least three independent experiments. Data were analyzed by unpaired *t*-test and ANOVAs. Data represent mean ± SD∗, p *< 0.05*，∗∗, p *< 0.01*，∗∗∗, p *< 0.001*, ∗∗∗∗, p *< 0.0001*.

To test whether neutrophils could move to immunization sites and correlate with the vaccine-induced antibody production, we prepared a vaccine rCPdA-E. The rCPdA-E is formulated by a recombinant capsid protein rCPdA from the porcine circovirus type 3 (PCV3) with an oil-in-water emulsion (E), so that we could detect the neutrophils in the immunization sites and in the draining lymph nodes (DLNs), as well as the levels of the antigen-specific antibodies in the sera of the mice immunized with rCPdA-E intraperitoneally (i.p.) or intramuscularly (i.m.) (Figure 1A). In this process, rCPdA and saline were used as controls. We found that the levels of the antigen-specific antibodies induced by rCPdA-E were approximately five times higher than that induced by its counterpart rCPdA in the sera of the mice either i.p. or i.m. immunized (Figure 1B), revealing the feasibility of using the oil-in-water emulsion to formulate the rCPdA in making the vaccines against the PCV3. Dynamic analysis of the neutrophil recruitment in immunization sites showed that after the initial i.p. immunization of mice with rCPdA-E, the percentages of Ly6G^+^ cells in the peritoneal lavage cells (PLCs) rose to about 5% at 1h, and it peaked at about 60% at 6h and then gradually decreased to about 50% at 12h (Figure 1C). Similarly, mice i.m. immunized with rCPdA-E had the most Ly6G^+^ cells in muscle tissues at 6h, accounting for about 25% (Figure 1D). Compared with the saline group, at 6h post-initial immunization, i.m./i.p. immunized mice with rCPdA or rCPdA-E had induced an 8% or 11% increase in neutrophils in the cells of the muscle tissue homogenate. Meanwhile, it induced a 40% or 60% increase in neutrophils in the PLCs, respectively (Figure 1E). It suggests that the number of the neutrophils recruited at the immunization site is positively correlated with the vaccine-induced antibody responses.

Considering that the neutrophils recruited at the immunization sites were reported to be able to take up antigens and then carry them to DLNs for assisting vaccine-induced antibody responses (Calabro et al., 2011, De Veer et al., 2013, Liang et al., 2017, Puga et al., 2011, Ohtsuka et al., 2001), we also detected neutrophils and antigen-bearing neutrophils in inguinal or mesenteric DLNs of immunized (i.m. or i.p.) mice. Unexpectedly, no obvious increase in neutrophils (Ly6G^+^) were detected in the DLNs isolated from the mice immunized with rCPdA or rCPdA-E by flow cytometry (Figure 1F, left; Figure S1A) and immunohistochemical staining (Figure 1F, right) and also no detectable antigen-bearing neutrophils (rCPdA^+^Ly6G^+^) in the DLNs (Figure S1B), indicating from another side that the place where the neutrophils recruited to the immunization site should be at the local in assisting vaccine-induced antibody responses.

### Neutrophil accumulation driven by peritoneal macrophages at immunization sites amplified the recruitment signaling and activated local and systemic B cells

(1) Neutrophil accumulation driven by peritoneal macrophages at immunization sites amplified the recruitment signaling and activated local B cells

**Figure 2:**
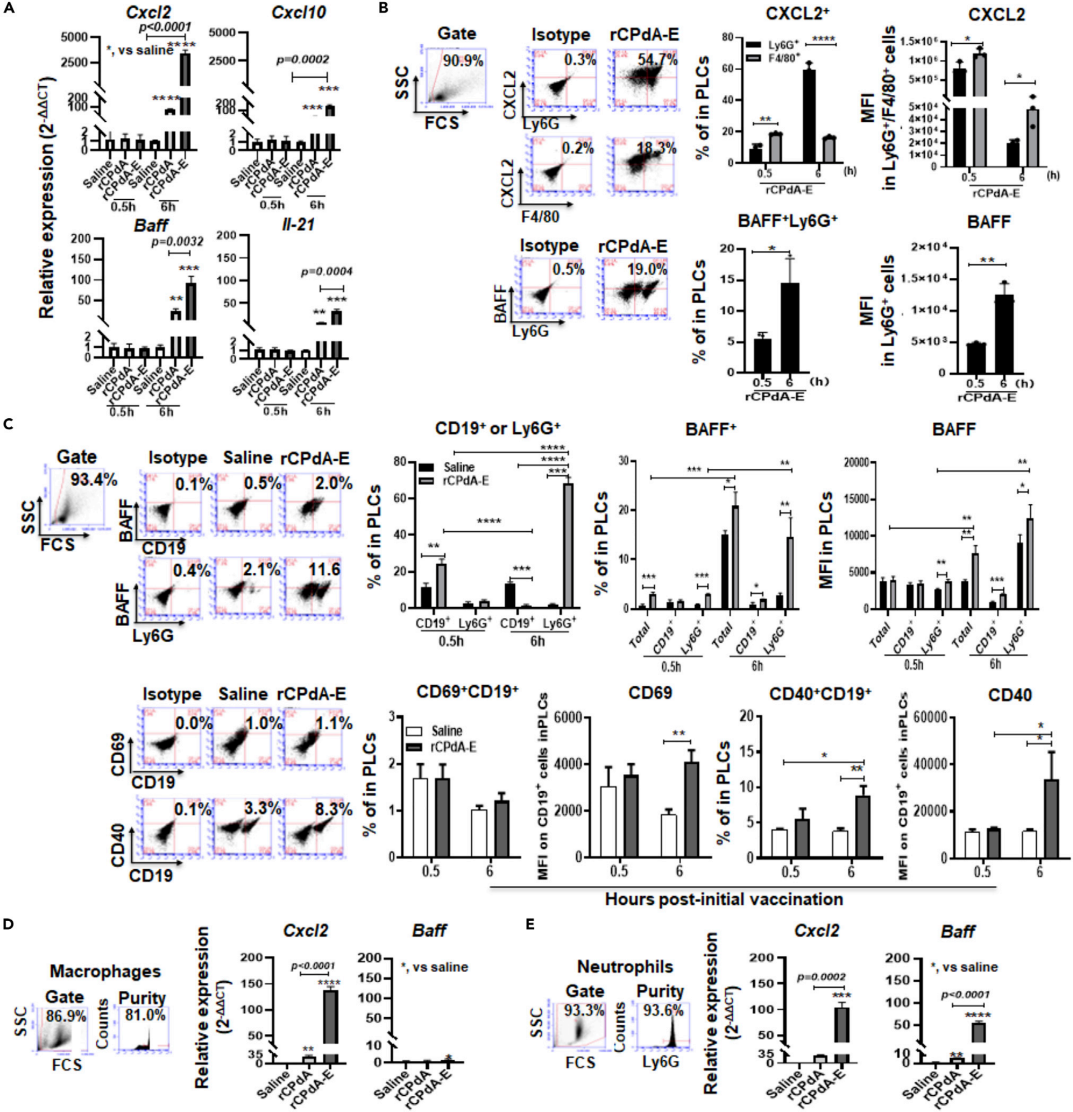
Relationship between neutrophils recruited to rCPdA-E immunization sites and the local macrophages and B cells Mice (n = 3 in each group) were i.p. immunized once on day 0, followed PLCs were collected at 0.5 and 6 h postimmunization and analyzed for the expression of chemokines and B cell activation factors and B cell activation by qRT-PCR or flow cytometry. Peritoneal macrophages and bone marrow neutrophils were isolated from naive mice and stimulated with rCPdA or rCPdA-E for 6 h followed by analyzing their expression of CXCL2 and BAFF mRNA by qRT-PCR. (A) The mRNA expression of neutrophil chemokines and B cell activation factors in PLCs. (B) CXCL2 and BAFF producing cells in PLCs. (C) The percentages of BAFF^+^Ly6G^+^, BAFF^+^CD19^+^ cells and the expression of CD69 and CD40 on CD19^+^ B cells in PLCs. (D) The mRNA expression of *Cxcl2* and *Baff* in cultured macrophages. (E) The mRNA expression of *Cxcl2* and *Baff* in cultured neutrophils. The data were the representative mean (mean ± SD) values of at least three independent experiments. Data were analyzed by unpaired *t*-test and ANOVAs. ∗, p *< 0.05*，∗∗, p *< 0.01*，∗∗∗, p *< 0.001*, ∗∗∗∗, p *< 0.0001*.

Because neutrophils can enter the immunization site, we urgently want to know where the driving and amplifying signals of neutrophil recruitment come from, and how the neutrophils recruited at the immunization site help in assisting the vaccine-induced antibody responses. Considering the convenience of obtaining cells from immunization sites, we chose i.p. immunized mice as the research object. We first detected the mRNA levels of chemokine *Cxcl2/10* and B cell activating factor (*Baff*)/*Il-21* in PLCs by qRT-PCR. Resultantly, the rCPdA-E immunization significantly upregulated the mRNA levels of *Cxcl2*, *Cxcl10*, *Baff,* and *Il-21* in the PLCs. Noticeably, the mRNA level of *Cxcl2* was 10-fold higher than that of *Cxcl10*, and the mRNA level of *Baff* was 2-fold higher than that of *Il-21* (Figure 2A). This result hinted that *Cxcl2* and *Baff* could be the major cytokines which are strongly involved in causing the vigorous neutrophil recruitment and promoting vaccine-induced antibody responses in mice immunized with rCPdA-E.

Tracing the CXCL2 and/or BAFF producing cells, we analyzed the PLCs using anti-F4/80 antibody (for recognizing macrophage), Ly6G antibody, and BAFF antibody in a flow cytometry assay. We found that at 0.5h after the rCPdA-E immunization, there were 9% of CXCL2^+^Ly6G^+^ cells and 19% of CXCL2^+^F4/80^+^ cells in the PLCs, and at 6h, these two kinds of CXCL2^+^ cells counted for 60 and 15%, respectively (Figure 2B, up). This indicates that the neutrophil recruitment to the immunization sites is initially driven by local macrophages and shortly afterward, majorly caused by the recruited neutrophils in a positive feedback way. In addition, we found that the recruited neutrophils could also produce high levels of BAFF in immunization sites as shown in Figure 2B. At 0.5 and 6 h post-rCPdA-E immunization, 5 and 14% of BAFF^+^Ly6G^+^ cells were detected in the PLCs, respectively. At 6h, the BAFF expression in Ly6G^+^ cells was 2.8-fold higher than that at 0.5h (Figure 2B, down).

Considering the existence of B cells, we kinetically analyzed the proportion and BAFF expression of the B cells and neutrophils in PLCs of the rCPdA-E immunized mice. We found that at 0.5h after rCPdA-E immunization, the proportion of CD19^+^ B cells in the PLCs was about 20%, whereas Ly6G^+^ cells were only 4.2%. However, at 6h, the proportion of Ly6G^+^ cells surged to about 70%, whereas the proportion of CD19^+^ B cells was only less than 1%. It should be noted that there were 4.2% of Ly6G^+^ cells recruited in the PLCs at 0.5h after rCPdA-E immunization among which there were 3% of BAFF^+^Ly6G^+^ cells, whereas there were no BAFF^+^ B cells at this time. At 6h of the immunization, in 20% more of BAFF^+^ PLCs, there were 15% of BAFF^+^Ly6G^+^ cells and less than 1% of BAFF^+^CD19^+^ cells, and the expression level of BAFF in Ly6G^+^ cells was 6-fold of that in CD19^+^ B cells (Figure 2C, up). Meanwhile, the levels of CD69 and CD40 on the PLCs doubled, and the proportion of CD40^+^CD19^+^ B cells increased from less than 5% to about 10% (Figure 2C, down). This suggested that the recruited neutrophils might be the earliest and the main BAFF-producing cells in immunization sites and possibly played an initiating role in assisting the local B cell activation.

To further confirm the CXCL2/BAFF producing cells induced by rCPdA-E, we cultured the peritoneal macrophages and bone marrow neutrophils that were isolated from the naive mice in the presence of rCPdA or rCPdA-E for 6h, and then analyzed their mRNA expression of *Cxcl2* and *Baff*. rCPdA-E induced a 5-fold higher expression of *Cxcl2* mRNA but barely increased the *Baff* mRNA in the macrophages (Figure 2D), whereas it induced a 3-fold and 10-fold higher expression of *Cxcl2* and *Baff* mRNA respectively in the neutrophils when compared with that induced by rCPdA (Figure 2E). Together, the *in vivo* and *in vitro* experiments reveal that macrophages at rCPdA-E immunization sites drive the recruitment of neutrophils, and the recruited neutrophils not only play a synergistic role with the macrophages but also initiate the activation of local B cells by producing a big amount of BAFF.

(2) Neutrophils recruited at the immunization sites activated systemic B cells by producing BAFF

**Figure 3:**
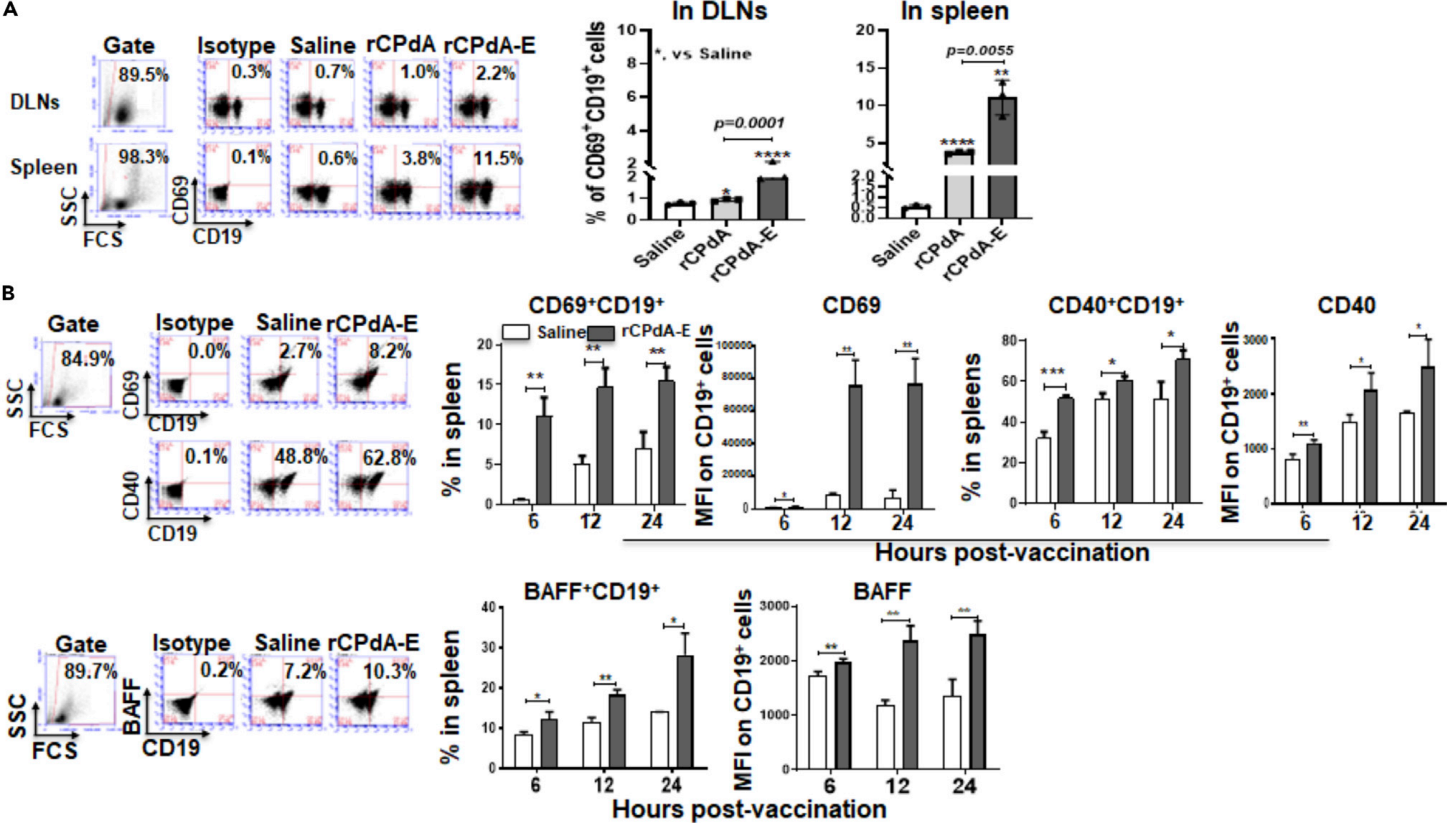
B cell activation and BAFF expression in lymph organs of immunized mice The spleens and DLNs were collected from the i.p. immunized mice (n = 3 in each group) for detecting B cell activation and BAFF expression by flow cytometry. (A) CD69 expression on B cells in DLNs and spleen at 6h. (B) The activation of splenic B cells at 6, 12, and 24 h. (C) BAFF-expressing splenic B cells at 6, 12, and 24 h. Student’s unpaired *t*-test was used to determine statistical significance of two groups and ANOVAs were used to determine multiple comparisons. Data represent mean ± SD p *< 0.05*，*∗∗,* p *< 0.01*，*∗∗∗,* p *< 0.001, ∗∗∗∗,* p *< 0.0001*.

To determine whether the neutrophil-derived BAFF could also function in an endocrine way and activate the systemic B cells, we checked the B cell activation in the DLNs and spleens of the mice i.p. immunized with rCPdA-E. At 6h post-immunization, we found that CD69^+^CD19^+^ B cells were increased about 2-fold in both of the DLNs and the spleens (Figure 3A). Kinetically, the percentage of CD69^+^CD19^+^/CD40^+^CD19^+^ cells were around 10 and 2 times higher in the spleens of mice immunized with rCPdA-E and the expression of CD69/CD40 on the CD19^+^ cells were continually increased in the spleens from 6h to 24h (Figure 3B), implying that the neutrophil-derived BAFF induced by rCPdA-E immunization at the immunization site (peritoneal cavity) could activate “remote” B cells in the DLNs and spleens. Furthermore, interestingly, BAFF^+^CD19^+^ cells also increased from 9% at 6h to 28% at 24h in the spleens of the mice (Figure 3C), hinting that the B cells, after being activated by the neutrophil-derived BAFF, might amplify their activation in an autocrine way.

(3) Neutrophils recruited at the immunization sites were required for local and systemic B cell activation

**Figure 4:**
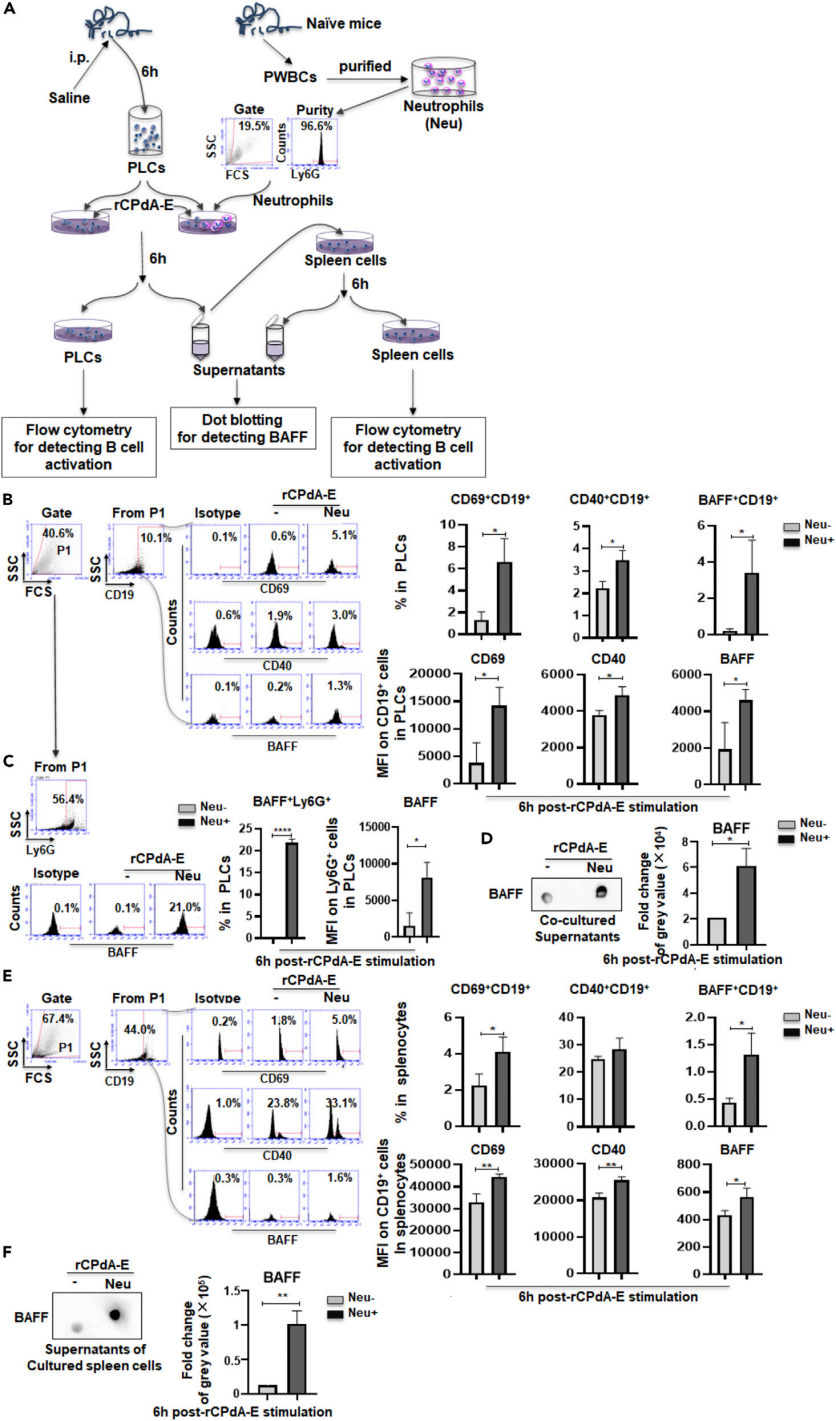
The effect of simulated neutrophil depletion on rCPdA-E induced B cell activation *in vitro* (A) Experiment procedure. (B) rCPdA-E-inducing the activation of B cells in PLCs of saline-injected mice with or without neutrophils purified from peripheral blood white cells (PWBCs) of naive mice. (C) Neutrophils added to the PLCs culture system produced BAFF under rCPdA-E stimulation. (D) The BAFF levels in the supernatant of the PLC culture system with rCPdA-E stimulation. (E) The induction role of supernatants of the PLC culture system on B cell activation of splenocytes. (F) The BAFF levels in the spleen cell culture supernatants. Student’s unpaired *t*-test was used to determine statistical significance of two groups. Data represent mean ± SD All the experiments were repeated three times. *∗,* p *< 0.05*, *∗∗*, p *< 0.01*, *∗∗∗*, p *< 0.001*, *∗∗∗∗*, p *< 0.0001*.

To confirm whether neutrophils recruited at immunization sites were required for local and systemic B cell activation after vaccine immunization — based on Figures 2C and S2A that neutrophils were barely detected in PLCs of mice injected with saline for 6h — we conducted an *in vitro* experiment in Figure 4A. Firstly, we examined the activation condition of the B cells in PLCs with or without the purified neutrophils (purity was 96.6% in Figure 4A) in the presence of rCPdA-E. The result showed that rCPdA-E could significantly upregulate the expression levels of CD69, CD40, and BAFF on/in the CD19^+^ B cells and boost the percentages of CD69, CD40, and BAFF expressing B cells in PLCs in the presence of neutrophils. Among them, in the PLCs, the percentages of B cells expressed by CD69 and BAFF increased 2–3 times compared with those counterparts without neutrophils, whereas the percentage of CD40^+^ B cells increased nearly 1-fold (Figure 4B). Because the presence of neutrophils seemed to be the deciding factor in terms of realizing the rCPdA-E-induced activation and BAFF expression of B cells in PLCs, we detected BAFF expression of the Ly6G^+^ neutrophils in the PLC culture system and found that about 23% of added Ly6G^+^ neutrophils (a half of total) could express BAFF under the stimulation of rCPdA-E (Figure 4C). Meanwhile, we also found that the BAFF level in the supernatant of the PLC culture systems with neutrophils tripled the one in which without neutrophils after rCPdA-E stimulation (Figure 4D). These results suggested that local B cell responses to vaccines relied on the presence of neutrophils, just like a recruitment of neutrophils *in vivo*. Secondly, we added the supernatant of rCPdA-E stimulated PLC culture system with or without neutrophils into the splenocytes of the mice to detect whether the presence of neutrophils is also necessary for the activation of systemic B cells (Figure 4A). We found that the supernatant with neutrophils could significantly upregulate the levels of CD69, CD40, and BAFF on/in CD19^+^ B cells by about 0.5-fold and the percentages of CD69^+^CD19^+^ and BAFF^+^CD19^+^ B cells by 1–1.5 times in splenocytes than those without neutrophils (Figure 4E). Moreover, stimulating by the supernatant of the PLC culture system, the secretion of BAFF in the splenocytes containing neutrophils was also significantly increased, which was about twice the amount of that without neutrophils (Figure 4F). These results suggested that the presence of neutrophils is also necessary for the vaccine-induced systemic B cell responses.

### rCPdA-E immunization activating the TLR9-IRF5 signaling of B cells in immunization sites and lymphoid organs

**Figure 5:**
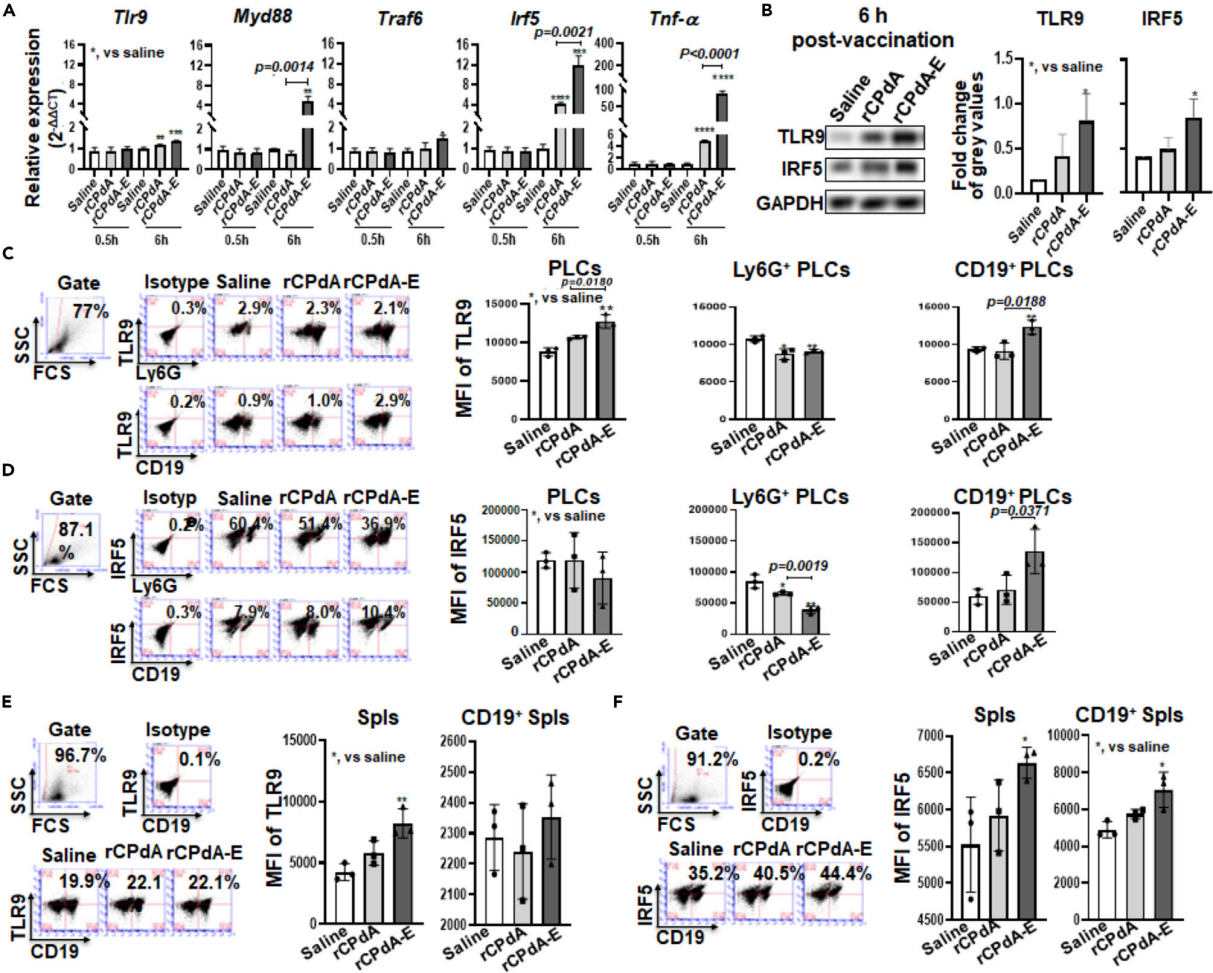
Expression of TLR9 and IRF5 in PLCs and spleens of immunized mice Mice (n = 3 in each group) were i.p. immunized with rCPdA or rCPdA-E once and then their PLCs and spleens were collected for detecting the expression of TLR9-IRF5 signaling molecules either by qRT-PCR at mRNA level or western blotting and flow cytometry at protein level. (A) The mRNA levels of TLR9-IRF5 signaling molecules in PLCs of the mice at 0.5 and 6h post-immunization. (B) The protein levels of TLR9 and IRF5 in PLCs of the mice at 6 h postimmunization. The expression of TLR9. (C) and IRF5. (D) in Ly6G^+^ and CD19^+^ PLCs of the mice at 6h post-immunization. The expression of TLR9. (E) and IIRF5. (F) in CD19^+^ splenocytes (spls) of the mice at 6h postimmunization. Student’s unpaired *t*-test was used to determine the statistical significance of two groups and ANOVAs were used to determine the multiple comparisons. Data represent mean ± SD All the experiments were repeated three times. *p< 0.05*，*∗∗,* p *< 0.01*，*∗∗∗,* p *< 0.001, ∗∗∗∗,* p *< 0.0001*.

To further explore how the rCPdA-E immunization helps inducing local and systemic B cell responses, we analyzed the mRNA expression of TLR9-IRF5 signaling molecules in PLCs of the i.p. immunized mice because of the study that the initiation of TLR9-IRF5 signaling pathway could make B cells more sensitive to BAFF (Abu-Rish et al., 2013; Giordano et al., 2020, Saurav De et al., 2018, Suthers and Sarantopoulos, 2017). As shown in Figures 5A and 5B, at 6h postimmunization, the mRNA expression of *Myd88*, *Irf5* and *Tnf-α* was increased about 6-fold, 4-fold, and 1-fold, and the protein levels of *Tlr9* and *Irf5* were both increased approximately 2 times in the PLCs, indicating an activation of the TLR9-IRF5 signaling pathway in immunization sites. Then, we analyzed the expression of TLR9 and IRF5 from the neutrophils and B cells in PLCs as well as that from the B cells in the spleens of the rCPdA-E immunized mice at 6h postimmunization. Resultantly, the TLR9 expression increased 1.4 times in CD19^+^ PLCs (Figure 5C) and tended to be upregulated in CD19^+^ spleen cells (Figure 5E). The IRF5 expression was significantly upregulated both in CD19^+^ PLCs (Figure 5D) and in CD19^+^ spleen cells (Figure 5F). The expression of TLR9 (Figure 5C) and IRF5 (Figure 5E) was barely upregulated in Ly6G^+^ PLCs. These results suggested that rCPdA-E immunization could activate the TLR9-IRF5 signaling pathway of the B cells in immunization sites and in lymphoid organs of the mice.

### Neutrophil-derived BAFF initiating B cell activation and antigen specific antibody responses

To measure whether the neutrophil-derived BAFF played an initial role in the vaccine-induced antibody responses, we tried to suppress the BAFF production using *Baff* siRNA *in vitro* and *in vivo* and then observed the changes happened in the B cell activation and antibody responses.

(1) *The in vitro experiment for determining the role of neutrophil-derived BAFF on initiating the activation of B cells*

**Figure 6:**
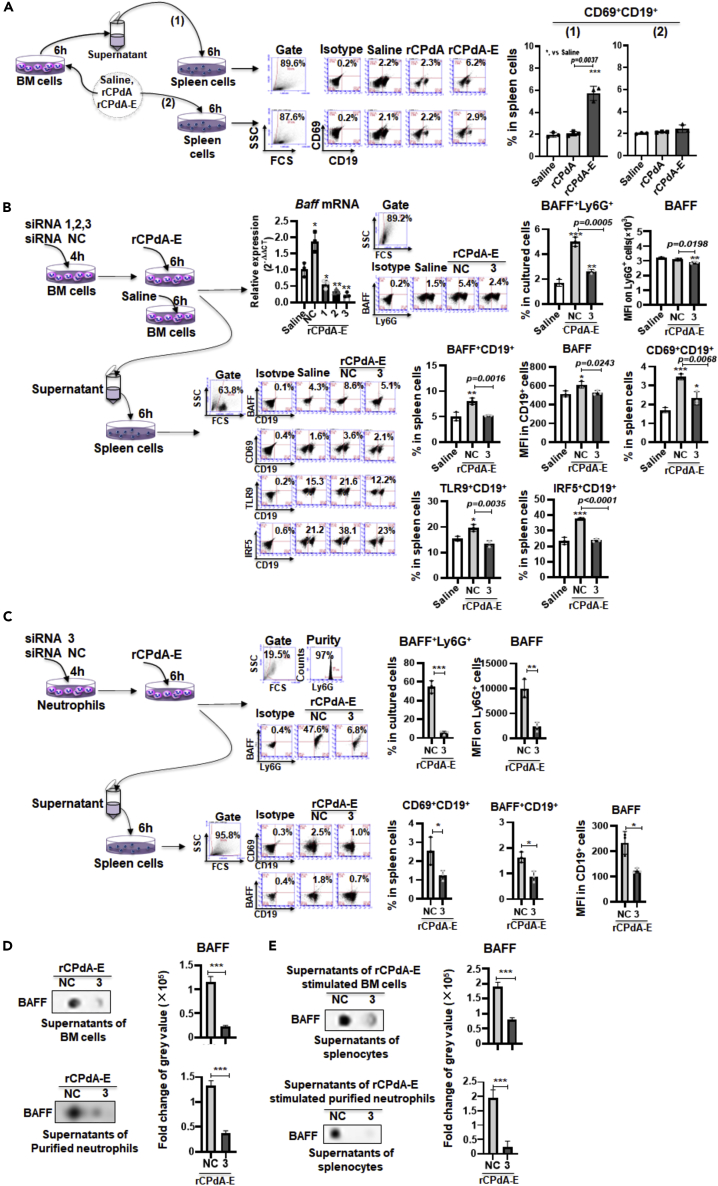
Effect of suppressing BAFF expression with *Baff* siRNA in neutrophils *in vitro* on B cell activation (A) The effect of rCPdA-E or its stimulated neutrophil-cultured supernatants on splenic B cell activation. (B) The effect of supernatants of neutrophils stimulated with rCPdA-E alone or plus *Baff* siRNA on splenic B cell activation. (C) The effect of supernatants of purified neutrophils stimulated with rCPdA-E alone or plus *Baff* siRNA on splenic B cell activation. (D) The levels of BAFF in the supernatants of BM cells or purified neutrophils stimulated by rCPdA-E with or without *Baff* siRNA-3. (E) The levels of BAFF in supernatants of splenocytes cultured with supernatants derived from rCPdA-E-stimulated BM cells or purified neutrophils with or without *Baff* siRNA-3. Student’s unpaired *t*-test was used to determine statistical significance of two groups and ANOVAs were used to determine multiple comparisons. Data represent mean ± SD All the experiments were repeated three times. *∗,* p *< 0.05*，*∗∗,* p *< 0.01*，*∗∗∗,* p *< 0.001*.

To determine whether the neutrophil-derived BAFF played a role on initiating the B cell activation, we cultured murine splenocytes *in vitro* for 6h in rCPdA or rCPdA-E containing medium or medium containing the supernatants of cultured murine bone marrow (BM) cells conditioned with rCPdA or rCPdA-E, respectively. Resultantly, the supernatants of rCPdA-E conditioned BM cells dramatically increased the percentage of CD69^+^CD19^+^ cells to about 2-fold in the spleen cells. However, it was not the case in the control group, where the rCPdA or rCPdA-E was added directly into the spleen cell culture (Figure 6A). It suggests that rCPdA-E can indirectly activate B cells by inducing BM cells to produce soluble B cell-activating factors.

We have proved that neutrophils in immunization sites could produce BAFF at a very early stage after rCPdA-E immunization. To verify whether the B cell activation was caused by neutrophil-derived BAFF, we used three *Baff* siRNAs named siRNA-1, siRNA-2, and siRNA-3 to interfere with BAFF expression in BM cells. We found that the siRNA-3 was the most effective one in suppressing the BAFF expression. Compared with siRNA-NC (Negative Control), siRNA-3 reduced the expression of *Baff* mRNA in Ly6G^+^ cells by 90%, meanwhile the percentage of BAFF^+^Ly6G^+^ cells decreased by 50% (Figure 6B, up). Then, we cultured murine splenocytes in the medium containing supernatants from neutrophil-enriched BM cells stimulated with rCPdA-E or rCPdA-E + siRNA-3 for 6h. We found that the rCPdA-E + siRNA-3 conditioned supernatants resulted in an obvious decrease in the percentages of BAFF^+^CD19^+^ and CD69^+^CD19^+^ cells from 8 to 3.5% in control group to 5 and 2.3% respectively (Figure 6B, middle). Similarly, the percentages of TLR9^+^CD19^+^ and IRF5^+^CD19^+^ cells in the spleen cells cultured in medium containing rCPdA-E + siRNA-3 conditioned supernatants declined from 20 to 37%–13 and 24%, respectively (Figure 6B, down).

Considering that BM cells also contain cells other than neutrophils, to determine whether neutrophils could assist the B cell response to vaccines, we purified neutrophils with the purity of 97% from BM cells and repeated the experiment as Figure 6B. We found the siRNA-3 treatment reduced the percentage of the BAFF^+^Ly6G^+^ cells by 10-fold in the rCPdA-E stimulated purified neutrophils. The supernatants from the purified neutrophils conditioned with rCPdA-E + siRNA-3 reduced the percentages of BAFF^+^CD19^+^ cells and CD69^+^CD19^+^ cells by 2-fold and 3-fold in the splenocytes, respectively (Figure 6C). Meanwhile, we also detected whether the BAFF levels in the supernatants from the Figures 6B and 6C related experiments were interfered by siRNA-3. Compared with siRNA-NC, siRNA-3 induced an obvious 4-fold decrease in BAFF levels in the supernatants of rCPdA-E cultured BM cells and purified neutrophils (Figure 6D). The siRNA-3 could also reduce the level of the BAFF in the supernatants of the splenocytes which were cultured in the medium containing supernatants of rCPdA-E-stimulated BM cells and purified neutrophils. The reduction was by 2-fold and 8-fold, respectively (Figure 6E). The *in vitro* results indicate that the BAFF suppression in neutrophils would lead to the repression of B cell activation.

(2) The *in vivo* experiment for verifying the initial role of neutrophil-derived BAFF in the vaccine-induced antibody responses

**Figure 7:**
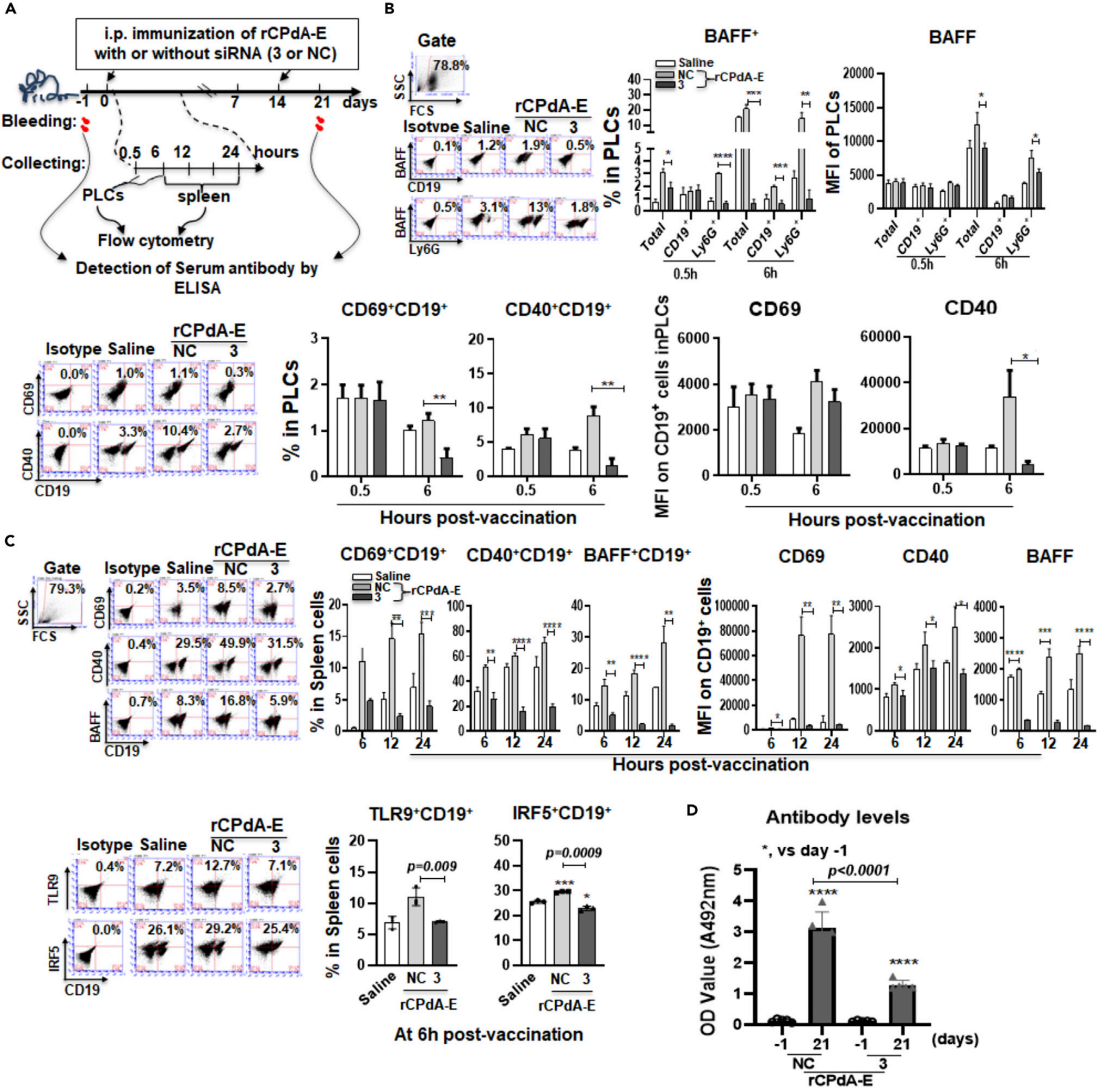
Effect of suppressing BAFF expression *in vivo* with *Baff* siRNA in the immunization site on B cell activation and vaccine-induced antibody production Mice (n = 3 in each group) were i.p. immunized using rCPdA-E plus *Baff* siRNA-3 or siRNA-NC, followed by collecting PLCs, spleens and sera to detect the BAFF expression and B cell responses, and antigen-specific antibody levels. (A) Experimental procedure. (B) Effect of interfering BAFF in PLCs. (C) Effect of interfering local BAFF in immunization sites on B cell responses in spleens. (D) Serum antigen-specific Antibody levels. Student’s unpaired *t*-test was used to determine statistical significance of two groups and ANOVAs were used to determine multiple comparisons. All the experiments were repeated three times. Data represent mean ± SD (n = 3). *p< 0.05*，*∗∗,* p *< 0.01*，*∗∗∗,* p *< 0.001, ∗∗∗∗,* p *< 0.0001*.

To measure whether the neutrophil-derived BAFF played an initial role in the vaccine-induced antibody responses, we examined the consequences of the BAFF suppression *in vivo*. Mice were i.p. immunized with rCPdA-E plus siRNA-3 or siRNA-NC, followed by detecting the BAFF expression and B cell activation in PLCs and spleens at different time post-initial immunization, and antigen specific antibody production in the sera of the mice at seventh day post-second immunization (Figure 7A). Our results showed that the production of BAFF by neutrophils in PLCs of mice i.p. immunized with rCPdA-E + siRNA-3 decreased significantly compared with that in mice immunized with rCPdA-E + siRNA-NC. The immunization with rCPdA-E + siRNA-3 decreased the percentage of BAFF^+^Ly6G^+^ cells in the PLCs from 3% to less than 1% at 0.5h, and from 20% to 1% at 6h, accompanied with the reduction of BAFF levels by 30% at 6h in the PLCs (Figure 7B, up). Meanwhile, the percentages of CD69^+^CD19^+^ and CD40^+^CD19^+^ cells in the PLCs also decreased from about 1 to 10% to less than 0.5 and 1%, respectively, and the CD40 levels on CD19^+^ cells reduced about 10 times at 6h after the immunization with rCPdA-E + siRNA-3 (Figure 7B, down). This reveals interfering neutrophil-derived BAFF can attenuate local B cell activation. By this inference, we would like to know whether the systemic immune responses induced by vaccines could also be attenuated by this effect. By detecting the activation of splenic B cells and the production of antigen-specific antibody in sera of mice immunized with rCPdA-E + siRNA-3, we found that the percentages of CD69^+^CD19^+^ cells, CD40^+^CD19^+^ cells, and BAFF^+^CD19^+^ cells and the expression levels of CD69, CD40, and BAFF on/in splenic B cells were remarkably reduced with the maximum decrease of about 20-fold and the minimum decrease of 1-fold at 6, 12, and 24 h (Figure 7C, up). Consistently, at 6 h postimmunization, rCPdA-E + siRNA-3 could reduce the splenic TLR9^+^CD19^+^ cells from 10% to 7% and IRF5^+^CD19^+^ cells from 40% to 23%, respectively (Figure 7C, down). Meanwhile, the immunization with rCPdA-E + siRNA-3 caused a reduction of vaccine-induced antibody levels in sera of the immunized mice by at least 1-fold (Figure 7D). These results demonstrate the suppression of BAFF in neutrophils at vaccine-immunization sites can reduce the systemic antibody responses by inhibiting the BAFF synthesis and the number of the activated B cells.

## Discussion

In this study, we observed that the immunization with rCPdA-E, a recombinant PCV3 capsid protein (rCPdA) formulated with an oil-in-water emulsion (E), triggered a massive amount of the neutrophils gathering in the immunization sites (up to 60%) and promoted the vaccine-induced antibody production, whereas in the DLNs the neutrophils and the antigen-bearing neutrophils were barely detected from the immunized mice. This seemed to indicate that neutrophils recruited to the immunization site had been participating in eliciting the B cell activation, which led to the production of higher antigen-specific antibodies. However, the results are inconsistent with the previous reports suggesting that massively recruited neutrophils induced by vaccines formulated with AS03 or MF59 around the immunization site take up antigens, and then migrate into the lymph nodes to promote the production of antigen-specific IgG by B cells (Calabro et al., 2011, De Veer et al., 2013, Liang et al., 2017, Puga et al., 2011, Ohtsuka et al., 2001). The discrepancy could be because of the difference of the oil phase used in the adjuvants. Our rCPdA-E was prepared using a mineral-oil-in-water emulsion adjuvant (Seppic, France), whereas03 and MF59 are squalene-based oil-in-water adjuvants. This reminds us that we can try using different adjuvants for comparative research in the future, which may provide valuable data in terms of developing more effective vaccines. Nevertheless, our results indicate that a large number of recruited neutrophils can play a role in promoting the production of vaccine-induced antibodies at the immunization site, which may account for the efficiency differences of the various vaccines.

However, instead of observing the reported phenomena again, we had revealed an alternative mode, in which the massively recruited neutrophils induced by rCPdA-E at the immunization site produced a high level of BAFF, and the neutrophil-derived BAFF in turn initiated the activation of the B cells in the local and lymphoid organs of the immunized mice. The *in vitro* experiment which simulates the neutrophil deletion *in vivo* had further implied that the neutrophil deficiency before vaccination compromised the responses of both the local and the systemic B cells to the vaccines. Remarkably, the BAFF level in the supernatant of cultured cells containing purified neutrophils indicated an abundant BAFF releasing, suggesting that neutrophil-associated BAFF release upon vaccine context is occurring through active BAFF secretion. This effect may occur by BAFF binding to BAFF receptor on B cells and activating the TLR9-IRF5 signaling in B cells, on account of that the BAFF receptor is only expressed on the B cells (Sevdali et al., 2021) while TLR9 is constitutively expressed in the B cells (Cunningham-Rundles et al., 2006). It is reported that the transmission of BAFF signal in B cells can promote the activation of TLR9 in B cells, which in turn enhance the expression of BAFF and induce the B cells to differentiate into plasma cells, so as to increase the level of antibody production (Abu-Rish et al., 2013, Giordano et al., 2020, Jackson and Davidson, 2019, Kumagai et al., 2008, Yang et al., 2019). Our results accorded with these reports, reflecting the mutually reinforcing feedback loop of BAFF and TLR9 in B cell activation. However, the obvious difference of our findings to the reports was that the feedback loop is not limited to the B cells in the lymphoid organs but also the ones in the local B cells. The BAFF expressed by recruited neutrophils in the immunization site could effectively initiate the signaling pathway of the TLR9-IRF5 in local B cells and prompt their BAFF expression, followed by amplifying the activation signals of the B cells in lymphoid organs. We verified the findings by using the RNAi assay with *Baff* siRNA to inhibit neutrophil-derived BAFF, causing the reduction in the number of the CD69^+^CD19^+^ and CD40^+^CD19^+^ B cells in the spleens and the drop of the vaccine-induced antibody level in the sera of the immunized mice. These results suggested that the neutrophils recruited to the immunization site played a role in promoting vaccine-induced antibody responses by generating BAFF to initiate local and systemic B cell responses and enhancing the TLR9-IRF5 signaling in B cells. Although there was no TLR9 agonist in rCPdA-E, we found the recruited neutrophils in PLCs could produce HMGB1 in addition to BAFF after the rCPdA-E i.p. immunization (Figure S2), which might be a source of TLR9 agonists (Bianchi et al., 2017; Liu et al., 2019; Yang et al., 2015) to activate the TLR9-IRF5 signaling pathway in the B cells.

Based on the results obtained in this study, we could illustrate the mode depicted in the Graphical Abstract. Specifically, rCPdA-E immunization initially activated residential macrophages to produce chemokines such as CXCL2, and then CXCL2 recruited the neutrophils from blood to the immunization site. The neutrophils that arrived were activated by rCPdA-E to release more CXCL2. Attracted by the abundant CXCL2, neutrophils swarmed into the immunization site upon the immunization, where they had been activated by rCPdA-E to produce BAFF and the other cytokines. The neutrophil-derived BAFF initiated the activation and the TLR9-IRF5 signaling pathway in the local B cells and brought about the subsequent enhancement of the systemic B cell responses and the vaccine-induced antibody production. Noticeably, TLR9 activation in B cells enhanced their BAFF production. Obviously, a positive feedback loop existed in the BAFF mediated B cell activation. Consequently, the neutrophil-derived BAFF activated the B cells to upregulate their CD40, enabling the B cells activate helper T cells to be more efficiently, and stimulated the B cells to differentiate into plasma cells, thus resulting in the enhanced antibody responses (Karmakar and Vermeren, 2021). Hopefully, by focusing on the research of the role of the neutrophil-driving BAFF at the immunization sites and the B cell activation at an early stage after vaccination could help us in evaluating the efficacy of the vaccines on time, instead of traditionally assessing the induction of memory B cells (Bmem) for long-term vaccine-induced protection (Ahmed and Slifka, 1998, Bernasconi et al., 2003, Mark et al., 2002, Traggiai et al., 2003, Yoshida et al., 2010, Yuichi Aiba et al., 2010).

### Limitations of the study

One limitation of this study was the lack of using different adjuvants for comparative research, which may otherwise provide valuable data for the future development of more effective vaccines. The other limitation was that we did not verify whether the rCPdA-E induced antibody responses could be reduced or even completely eliminated if we interfered with the HMGB1 expression in the recruited neutrophils, and we would like to find out that in the future. We also did not prove that either HMGB1 or BAFF expressed earlier in the neutrophils after the rCPdA-E immunization. According to our results obtained so far, we could find that there was 15–20% of BAFF^+^Ly6G^+^ cells and 10% of HMGB1^+^Ly6G^+^ cells in the PLCs of the immunized mice at 6 h after the rCPdA-E immunization; this might suggest that neutrophil-derived BAFF played a dominant role in initiating B cell responses, which was enhanced by HMGB1.

## STAR★Methods

### Key resources table

**Table:** 

REAGENT or RESOURCE	SOURCE	IDENTIFIER
**Antibodies**		

Mouse monoclonal to TLR9	Abcam	Cat#ab134368
Mouse monoclonal to IRF5	Abcam	Cat#ab33478
Goat anti-mouse IgG	Proteintech	Cat#SA00001-1
Goat anti-rabbit IgG	Proteintech	Cat#SA00001-2
Rat Anti-Mouse CD19	BD	Cat#557399
Rat Anti-Mouse CD69	BD	Cat#553236
Rat Anti-Mouse CD40	BD	Cat#561845
Rat Anti-Mouse F4/80	BD	Cat#565410
Rat Anti-Mouse Ly6G	BD	Cat#560599
Rat Anti-Mouse IRF5	Biolegend	Cat#158604
Rabbit Anti-Mouse BAFF	Biorbyt	Cat#orb495712
Rabbit Anti-Mouse CXCL2	Biorbyt	Cat#orb10749
Rabbit polyclonal to HMGB1	Abcam	Cat#ab18256
Goat Anti-Rabbit IgG H&L (Alexa Fluor® 488)	Abcam	Cat#ab150077
Goat Anti-Rabbit IgG H&L (APC)	Abcam	Cat#ab130805

**Chemicals, peptides, and recombinant proteins**		

Histopaque-1119	SIGMA	Cat#11191
Histopaque-1077	SIGMA	Cat#10771

**Oligonucleotides**		

Specific primers for target genes (Table 1)	This paper	N/A

**Software and algorithms**		

GraphPad Prism	GraphPad	https://www.graphpad.com/scientific-software/prism/
ImageJ	softonic	https://imagej.en.softonic.com/
Accuri C6	BD Biosciences	N/A

### Resource availability

#### Lead contact

Further information and requests concerning the resources and reagents please send to the lead contact, Liying Wang (wangliy@jlu.edu.cn).

#### Materials availability

This study did not generate new reagents.

### Experimental model and subject details

#### Animals

Female outbreeding strain ICR mice (6–8 weeks) were purchased from the Yisi Laboratory Animal Technology Co., Ltd., Changchun, China, and maintained in specific pathogen-free conditions in the Laboratory Animal Center of Jilin University. The experimental manipulation of the mice was undertaken in accordance with the National Institute of Health Guide for the Care and Use of Laboratory Animals, with the approval of the Scientific Investigation Board of Science & Technology of Jilin Province, China. And the mouse experiments were approved by the ethics committee of the College of Basic Medical Sciences of Jilin University with the number of 2020–94.

### Method details

#### Vaccine preparation and immunization

rCPdA-E, a vaccine composed of rCPdA (5μg/mouse) and an oil-in-water emulsified adjuvant (E, Seppic®, France) in a 3:1 ratio (antigen volume: adjuvant volume), was used to immunize mice. rCPdA was a His-tagged recombinant capsid protein (rCP) of porcine circovirus type 3 (PCV3), expressed in the *E. coli* system and purified to a purity over 90%.

In order to test the vaccine-induced antibody production, mice were immunized with rCPdA or rCPdA-E (100μL/mouse) intraperitoneally (i.p.) or intramuscularly (i.m.) on day 0 and 14. And then bled on day -1, 21 or 28 for detecting serum antigen-specific antibody levels by ELISA. In this process, saline injection mice group was taken as a negative control. For detecting neutrophil recruitment and B cell activation, the PLCs, DLN cells and spleen cells were collected at different time points after immunization. All of the cells were prepared for subsequent experiments.

#### Cells isolation and culture

To detect CD40^+^ B cells, CD69^+^ B cells, neutrophils and antigen-bearing neutrophils in the DLNs, we collected inguinal lymph nodes on the same side of the i.m. immunized mice and the mesenteric lymph nodes of the i.p. immunized mice. The lymph node cells were released by grinding in 1mL RPMI 1640 containing 10% (V/V) FBS in a sterile Petri plate, filtered through a 300-mesh filter, centrifuged at 600g for 5 min and then resuspended with PBS containing 2% FBS (FBS-PBS). The cells were counted by flow cytometry and used for detecting the neutrophils and the B cells.

To isolate the neutrophils from the bone marrow (BM) of the naïve mice, the femur and tibia were removed from the immunized mice. The epiphyses of the bones were cut off, and BM cells were flushed using a 25-gauge needle and a 12cc syringe with RPMI 1640 supplemented with 10% (V/V) fetal bovine serum (GIBCO) (FBS) and 2Mm EDTA. The BM neutrophils were separated on a density gradient centrifugation using Histoque-1119 and 1007.

To extract the resident peritoneal macrophages in the PLCs of naïve mice, we pulled up the peritoneal membrane using a forceps and using a 25-gauge needle injected 6mL of ice-cold PBS with 2% (V/V) FBS in the peritoneal cavity. We shaked the mouse for 10s to detach macrophage from the peritoneal cavity and get a cell suspension. Then, using a 21 G needle in a 10 mL syringe, we removed the macrophages suspension from the peritoneal cavity. Collected the cell suspension in a 15 mL tube containing and placed on ice. Centrifuged the cells suspension at 350 g at 4°C for 5 min. Resuspended the cell pellet with RPMI 1640 supplemented with 10% of FBS and incubated for 2h at 37°C, then washed off the unattached cells with PBS at 37°C. Added RPMI 1640 with 10% FBS to the attached macrophages and proceeded with the experiments. This protocol was referenced by the other researchers (Rios et al.,2017).

To obtain splenocytes, we grinded spleens of naïve mice in 5mL ice-cold RPMI 1640 with 10% (V/V) FBS, then filtered through a 300-mesh filter into a 5mL centrifuge tube on ice. Centrifuged the cells suspension at 350 g at 4°C for 5 min. Resuspended the cell pellet to lyse the red blood cells using 3mL ACK buffer (1/9 v/v) (NH_4_Cl 8.024 mg/L, KHCO_3_ 1001 mg/L, Na_2_EDTA 3.7 mg/L, pH 7.2–7.4) for 7–10 min, followed by washing and centrifugation steps repeatedly. Resuspended the cell pellet with RPMI 1640 supplemented with 10% of FBS. Counted the cells, plated them at 1×10^6^ cells/mL and incubated for 2h at 37°C and proceeded with the experiments.

For detecting the effect of BAFF expression in neutrophils on B cell activation, the isolated BM neutrophils (4×10^6^ cells/mL) were maintained in RPMI 1640 supplemented with 10% FBS and antibiotics, seeded in a 24-well plate (Costar, Cambridge, MA), and cultured with 2.5μg/mL PCV3-rCP, 3μL/mL emulsion or equal volume saline for 0.5 or 6h in a 5% CO_2_-humidified incubator at 37°C. Centrifuged at 750 g for 5 min at 4°C, the cultured neutrophils were collected for further using, and the supernatant was collected for stimulating splenic B cells. 6h later, the activation of the splenic B cells was analyzed.

#### Western blotting and dot blotting

For Western Blotting, the PLCs of the immunized mice were lysed in ice-cold RIPA buffer containing 0.1mM PMSF. The lysates were quantified using a BCA protein assay kit (CW0014, CW Biotech). The lysates from the tissues were blotted using PVDF membranes (Millipore, Billerica, MA, USA). The membranes were blocked for 2h at room temperature (22–25 °C) with PBS containing 5% dried nonfat milk and probed with anti-TLR9 antibodies (clone 26C593.2; Abcam) anti-IRF5 antibodies (clone 10T1; Abcam) at 4 °C overnight. After being washed with PBST three times at 5min intervals, membranes were then incubated with horseradish peroxidase-conjugated goat anti-mouse IgG (SA00001-1, Proteintech) for 1h at room temperature.

For Dot Blotting, the PLC-free lavage fluid of immunized mice was quantified using BCA protein assay kit, followed by blotting using PVDF membranes. As mentioned above, the membranes were blocked, probed with anti-HMGB1 antibodies (ab18256, Abcam) monoclonal antibodies, washed, and incubated with horseradish peroxidase-conjugated goat anti-rabbit IgG (SA00001-2, Proteintech).

After being further washed, the membranes were treated with EasySee Western Blot Kit (TransGen Biotech, DW101-02) and the immunoreactive bands were visualized by Amersham Biosciences Hyperfilm ECL (GE Healthcare Life Sciences).

#### Indirect enzyme-linked immunosorbent assay (I-ELISA)

The rCPdA (0.2μg/well) was coated on 96-well polystyrene microtiter plates and incubated overnight at 4°C in 0.01 mol/L PBS (1 mmol/L KH_2_PO_4_, 10 mmol/L Na_2_HPO_4_, 137 mmol/L NaCl, 2.7 mmol/L KCl, pH 7.4). After washing for three times with washing buffer (PBST, 0.01 mol/L PBS, 0.05% Tween 20), 200μL of blocking buffer was added (0.01 mol/L PBS, 5% skim milk) followed by incubating at 37°C for 2h. The sera were diluted at 1:5000 with sample buffer (0.01 M PBST, 5% skim milk), added to wells in duplicate and incubated at 37°C for 1h. Afterwards, plates were washed three times followed by the addition of 100μL per well of goat anti-mouse IgG (SA00001-1, Proteintech) at 1:5000 dilution and incubation at 37°C for 1h. After washing three times, the substrate solution of o-phenylenediamine dihydrochloride (OPD) (Amresco) was added and incubated at room temperature for 5 min for color development which was stopped with 50μL per well of 2 M sulphuric acid. The optical density (OD) of the color in each well of plates was determined at 492 nm on an automated ELISA plate reader. The results were expressed as A492 ± SD.

#### Histological analysis and immunohistochemistry

The lymph nodes of the injected mice were fixed in 4% (w/v) paraformaldehyde, embedded into paraffin, and cut into 4μm thick sections. The sections were H&E stained and observed under the microscope. For immunohistochemistry (IHC), the tissue sections were dewaxed, rehydrated and then incubated in citrate buffer at 95°C for 15 min, enabling antigen retrieval. After being treated with hydrogen peroxide for 10 min at room temperature, the sections were incubated with 1:50 diluted anti-Ly6G antibodies (clone 1A8; BD) at room temperature for 2h and then washed, followed by incubation with horseradish peroxidase–streptavidin complex (Maixin, Fuzhou, China) for 30 min at 37°C. The sections were then stained with DAB (Maixin) and counterstained with hematoxylin. All steps were performed at room temperature unless otherwise specified. Pathological scores of the tissues were determined based on the criteria: 0, no neutrophils; 1, few neutrophils (<25%); 2, moderate neutrophils (25–50%); 3, many neutrophils (>50%).

#### Flow cytometry

For direct and surface staining, the isolated cells were stained with the fluorescence conjugated monoclonal antibodies against mouse Ly6G (clone 1A8; BD), CD19 (clone 1D3; BD), CD69 (clone H1.2F3; BD), CD40 (clone 3/23; BD), F4/80 (clone T45-2342; BD), which were purchased from BD Biosciences, at 4°C in dark for 30 min.

For indirect intracellular staining and detecting His-tagged antigen uptake, the cells were first fixed with 4% paraformaldehyde and permeabilized with 0.1% saponin for 10 min, then blocked with the normal serum (5% v/v) from the host species for 30 min, followed by staining with anti-HMGB1 (ab18256, Abcam), anti-CXCL2 (orb10749, Biorbyt) and anti-His-tag (ab9108, Abcam) monoclonal antibodies for 30 min. After being washed twice with FACS buffer (PBS containing 0.5% BSA and 2 mM EDTA), incubated the cells with Alexa Fluor 488-conjugated goat-anti-rabbit (ab150077, Abcam) or APC-conjugated goat-anti-rabbit (ab130805, Abcam) secondary antibody for 30 min. For direct intracellular molecule staining, the fixed and permeabilized cells were stained with anti-TLR9 (clone 1138D; R&D), anti-IRF5 (clone W16007B; Biolegend) and anti-BAFF (orb495712, Biorbyt) antibodies at 4°C in dark for 30 min.

Following by washing twice with 2 FACS buffer, the cells were resuspended in PBS and analyzed by Accuri C6 (BD Biosciences). Live cells were carefully gated by forward and side scattering.

#### RNA isolation and quantitative real-time PCR analysis

Total RNA was isolated from the samples with Trizol (CWBIO, CW0580S) and reverse transcribed using cDNA Synthesis Kit (TransGen Biotech, Beijing, China). Quantitative real-time polymerase chain reaction (qRT-PCR) was performed using two-step SYBR green qPCR assays and the target genes were amplified with the following specific primers (Table 1). The forward and reverse primers were designed to be present in different exons of the targeted transcript to prevent amplification of genomic DNA. The data were acquired using the Step One™ real-time PCR system (Applied Biosystems, Foster City, CA, USA). The procedures of the target mRNA amplification were as follows: one cycle at 95°C (30 s) followed by 40 cycles at 95°C (5 s) and 64°C (31 s). Each assay plate included negative controls with no template. The mRNA levels were normalized with the mRNA levels of β-actin and analyzed with 2^-ΔΔCt^ method.

**Table 1:** The sequence of specific primers

Gene	Sequence	
*β-actin*	5′-GATCAAGATCATTGCTCCTC-3′	5′-AGGGTGTAAAACGCAGCTCA-3′
*Baff*	5′-GTTCCATGGCTTCTCAGCTT-3′	5′-TTCCTCTGGATGACATGACC-3′
*Il-21*	5′-AAACTCAAGCCATCAAACCC-3′	5′-AAGGGCATTTAGCTATGTGC-3′
*Cxcl2*	5′-GCGCCCAGACAGAAGTCATA-3′	5′-CGAGGCACATCAGGTACGAT-3′
*Cxcl10*	5′-CTGCCCACGTGTTGAGATCAT-3′	5′-AGAGGCTCTCTGCTGTCCAT-3′
*Tnf-α*	5′-CCTGTAGCCCACGTCGTAG-3′	5′-GGGAGTAGACAAGGTACAACC-3′
*Tlr9*	5′-ACCCTGGAAGAGCTAAACCTG-3′	5′-CAGTTGCCGTCCATGAATAGG-3′
*Myd88*	5′-TCGCAGTTTGTTTGCCTG-3′	5′-TGTAAAGGCTTCTCGGACTCC-3′
*β-actin*	5′-TCGCAGTTTGTTTGCCTG-3′	5′-TGTAAAGGCTTCTCGGACTCC-3′
*Baff*	5′-AGCGGGAAGTCAAGACGAAG-3′	5′-CTGAGAACATCTCCAGCAGC-3′

Related to Figures 2, 5, and 6.

#### RNA-mediated interference

For RNAi experiments *in vivo*, mice were i.p. immunized with rCPdA-E contained *Baff* siRNA-3 or siRNA-NC 5μg/mouse.

For each RNAi assay *in vitro*, the isolated neutrophils were spread with Opti-MEM in a 24-well plate at a concentration of 3×10^6^ cells/well. For each well sample, prepare the mixture of 3μL transfection reagent (GenePharma, Shanghai, China) with 25μL Opti-MEM; use 25μL Opti-MEM to dilute siRNA synthesized by GenePharma (Shanghai, China) to a final working concentration of 100 nM. Gently mix the transfection reagent and diluted siRNA, and put it at room temperature for 15 min. Then, drip the mixture into the well plate. Shake the plate to increase the chance of cells to contact with siRNA. After 4h of transfection, discard the transfection supernatant and add 1mL of RPMI 1640 containing 10% FBS to do the latter study. The mouse-specific sense sequences of siRNAs targeting *Baff* were as follows: siRNA-NC 5′-UUCUCCGAACGUGUCACGUTT-3′, 5′-ACGUGACACGUUCGGAGAATT-3′; *Baff* siRNA-3 5′-GCAACGGAGACGACACCUUTT -3′, 5′-AAGGUGUCGUCUCCGUUGCTT -3′.

### Quantification and statistical analysis

Data are showed as mean ± SD. All calculations and statistical analyses were performed using GraphPad Prism 8.0 for Windows (San Diego, CA). Student’s unpaired t-test was used to determine the statistical significance of the two groups. For multivariate data analysis, ANOVAs were used instead. p value of less than 0.05 (95% CI) was considered to be statistically significant. All *in vivo* and *in vitro* experiments were performed at least three times.

## Data Availability

Data reported in this paper will be shared by the lead contact upon request. This paper does not report original code. Any additional information required to reanalyze the data reported in this paper is available from the lead contact upon request.
