# Supplementary material for: Neutrophils recruited to immunization sites initiating vaccine-induced antibody responses by locally expressing BAFF
Source: iScience. 2022 May 23;25(6):104453. doi: 10.1016/j.isci.2022.104453 (PMC9301880; doi:10.1016/j.isci.2022.104453)
Supplement: Document S1. Figures S1 and S2 [file mmc1.pdf]

iScience, Volume *25*

Supplemental information

Neutrophils recruited to immunization sites initiating vaccine-induced antibody responses by locally expressing BAFF

Yangyang Wang, Kuo Qu, Wenting Lu, Peiyan Zhao, Zhe Wang, Cuiyun Cui, Ye Liu, Ming Yang, Yongli Yu, and Liying Wang

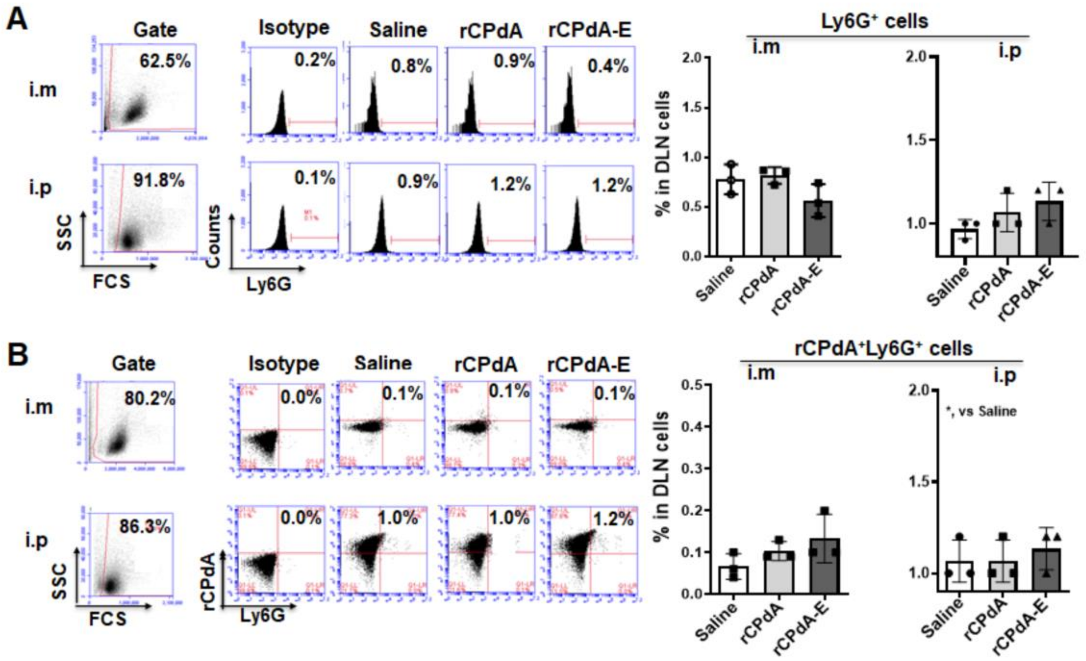

Figure S1. The percentages of neutrophils and antigen-bearing neutrophils in DLNs of immunized mice, Related to Figure 1. Mice (n=3 in each group) were immunized i.m. or i.p. with rCPdA or rCPdA-E on day 0. The $Ly6G^{+}$ neutrophils or $rCPdA^{+}Ly6G^{+}$ neutrophils were detected in the DLNs of immunized mice by flow cytometry. For detecting $rCPdA^{+}Ly6G^{+}$ neutrophils, DLN cells were isolated from inguinal lymph nodes or mesenteric lymph nodes of i.m. or i.p. immunized mice, fixed, permeabilized, and then recognized by anti-His-tag antibody and Ly6G antibody, followed by a flow cytometry assay. (A) The percentages of $Ly6G^{+}$ neutrophils in DLNs. (B) The percentages of $rCPdA^{+}Ly6G^{+}$ neutrophils in DLNs. The data were the representative mean values of three independent experiments. Data were analyzed ANOVAs for multiple comparison test. * , $p<0.05$， **, $p<0.01$， ***, $p<0.001$, ****, $p<0.0001$.

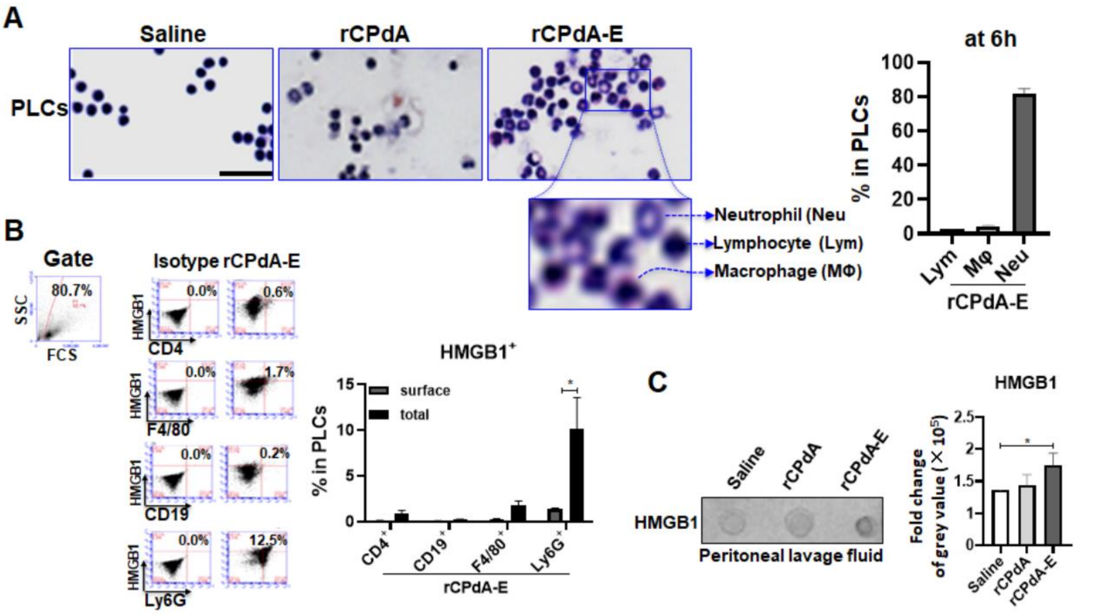

Figure S2. rCPdA-E induced HMGB1 expression in murine PLCs, Related to Figure 4. Mice (n = 3 in each group) were i.p. injected with rCPdA, rCPdA-E or saline once. 6h later, the lavage was collected and analyzed for the morphology of PLCs by H&E staining, HMGB1 expression either in PLCs or different type cells of PLCs by flow cytometry and the HMGB1 level in PLC-free lavage fluid by dot blotting. **(A)** Morphology of PLCs and percentage of different cells in PLCs. Scale bars represent 50 μm in length. **(B)** Percentages of different $HMGB1^{+}$ cells in PLCs. **(C)** HMGB1 in the PLC-free lavage fluid. Student's unpaired t-test was used to determine statistical significance of two groups and ANOVAs were used to determine multiple comparisons. All the experiments were repeated three times. Data represent mean ± SD (n=3). *p<0.05*， **, *p<0.01*， ***, *p<0.001*, ****, *p<0.0001*.
